# Supplementary material for: Silibinin Overcomes EMT-Driven Lung Cancer Resistance to New-Generation ALK Inhibitors
Source: Cancers (Basel). 2022 Dec 11;14(24):6101. doi: 10.3390/cancers14246101 (PMC9777025; doi:10.3390/cancers14246101)
Supplement: Supplementary file 1 [file cancers-14-06101-s001.zip › cancers-2047282-supplementary.pdf]

Silibinin overcomes EMT-driven lung cancer resistance to new-generation ALK inhibitors

SUPPLEMENTARY INFORMATION

Table S1. Comparison of ALK-TKIs IC_{50} values (in μmol/L) in ALK-rearranged NSCLC cells cultured in the absence/presence of SBN

	H2228	H2228/CR	H3122	H3122/CR
**crizotinib**	2.1 ± 0.8 (1.0)	10.6 ± 1.7 (1.0)	0.29 ± 0.06 (1.0)	1.08 ± 0.40 (1.0)
+ 100 μmol/L SBN	1.3 ± 0.7 (1.6)	3.7 ± 0.7 (2.9)[a]	0.10 ± 0.00 (2.9)	0.17 ± 0.03 (6.3)
**brigatinib**	0.5 ± 0.08 (1.0)	3.8 ± 0.4 (1.0)	0.02 ± 0.00 (1.0)	0.12 ± 0.03 (1.0)
+ 100 μmol/L SBN	0.4 ± 0.07 (1.2)	0.6 ± 0.1 (6.3)	0.01 ± 0.00 (2.0)	0.07 ± 0.01 (1.7)
**lorlatinib**	1.0 ± 0.11 (1.0)	83 ± 5.1 (1.0)	0.004 ± 0.00 (1.0)	0.02 ± 0.00 (1.0)
+ 100 μmol/L SBN	9.0 ± 2.2 (0.1)	19.3 ± 3.2 (4.3)	0.004 ± 0.00 (1.0)	0.003 ± 0.00 (6.7)

[a] Number in parentheses represent the silibinin (SBN) extent of sensitization expressed as fold-change compared to untreated control cells

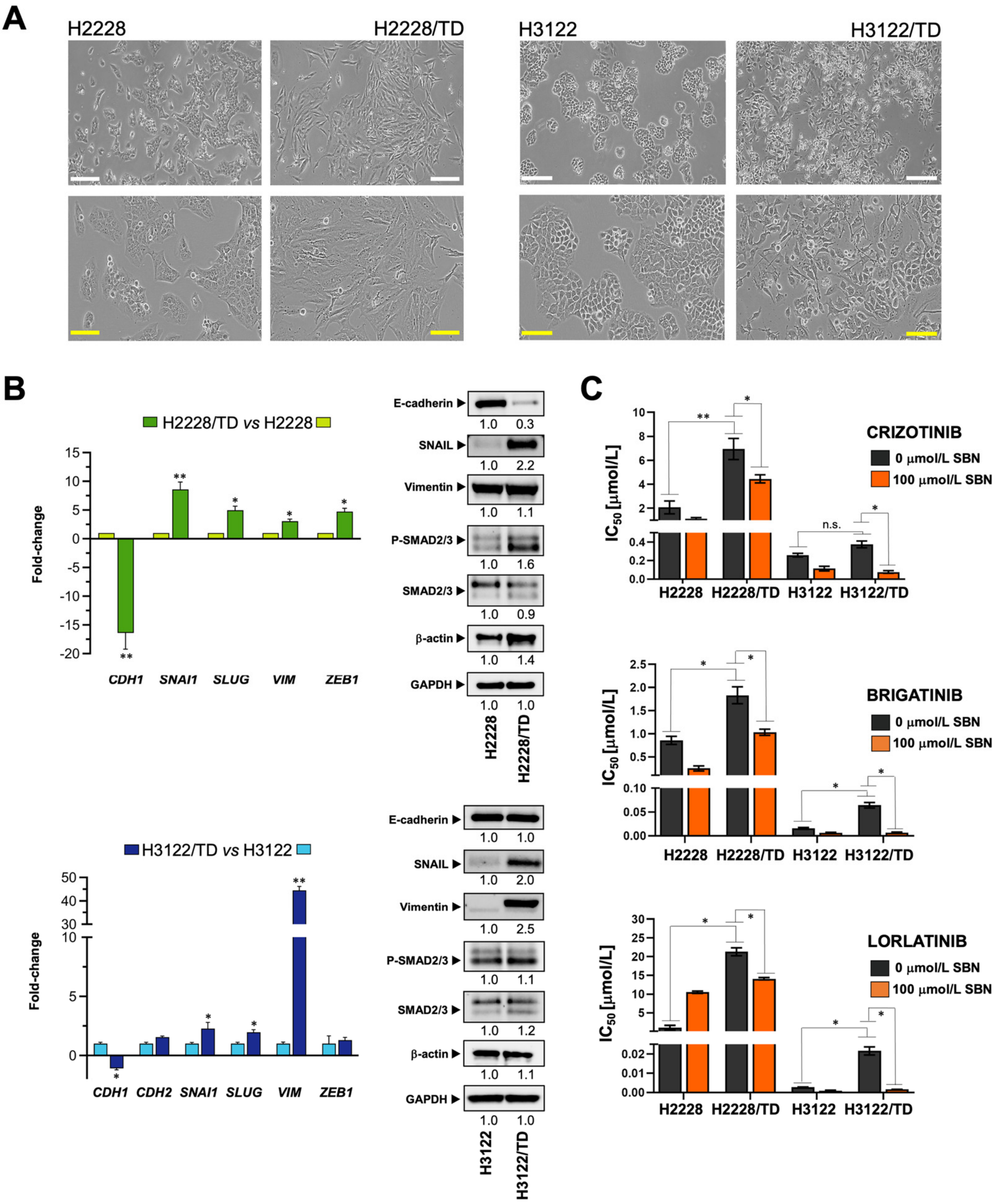
A
H2228
H2228/TD
H3122
H3122/TD
B
H2228/TD vs H2228
Fold-change
CDH1
SNAI1
SLUG
VIM
ZEB1
E-cadherin
SNAIL
Vimentin
P-SMAD2/3
SMAD2/3
β-actin
GAPDH
H3122/TD vs H3122
CDH2
C
CRIZOTINIB
BRIGATINIB
LORLATINIB
0 μmol/L SBN
100 μmol/L SBN
IC50 [μmol/L]
n.s.

Figure S1. EMT caused by chronic TGFβ stimulation drives cross-resistance to multiple-generation ALK-TKIs. A. Representative phase contrast microphotographs of H2228/H2228TD and H3122/H3122TD ALK-rearranged NSCLC cell line pairs. TD: transdifferentiated; Scale bar, 100 μm. **B.** *Left.* The transcript abundance of *CDH1*, *CDH2*, *SNAI1*, *SLUG*, *VIM*, and *ZEB1* was calculated using the ΔC_t method and presented as fold-change in H2228/H2228TD and H3122/H3122TD cells; *p<0.05 and **p<0.005, statistically significant differences. *Right.* Expression levels of E-cadherin, SNAIL, vimentin, phospho-SMAD2/3, total SMAD2/3 were detected by immunoblotting in H2228/H2228TD and H3122/H3122TD cells using specific antibodies. The intensity of the bands was measured using the ImageJ software. Fold-change of each protein relative to parental cells was calculated using GAPDH as a loading control. Figure shows representative immunoblots of multiple (*n*=3) independent experiments. **C.** Bar graphs showing the MTT-based IC_{50} values of crizotinib, brigatinib, and lorlatinib for H2228/H2228TD and H3122/H3122TD cells calculated in the absence or presence of 100 μmol/L silibinin. The results are presented as the means (*columns*) ± S.D (*bars*) (n=3, in triplicate). *p<0.05 and **p<0.005, statistically significant differences; n.s. not significant.

Figure S2. Uncropped western blots

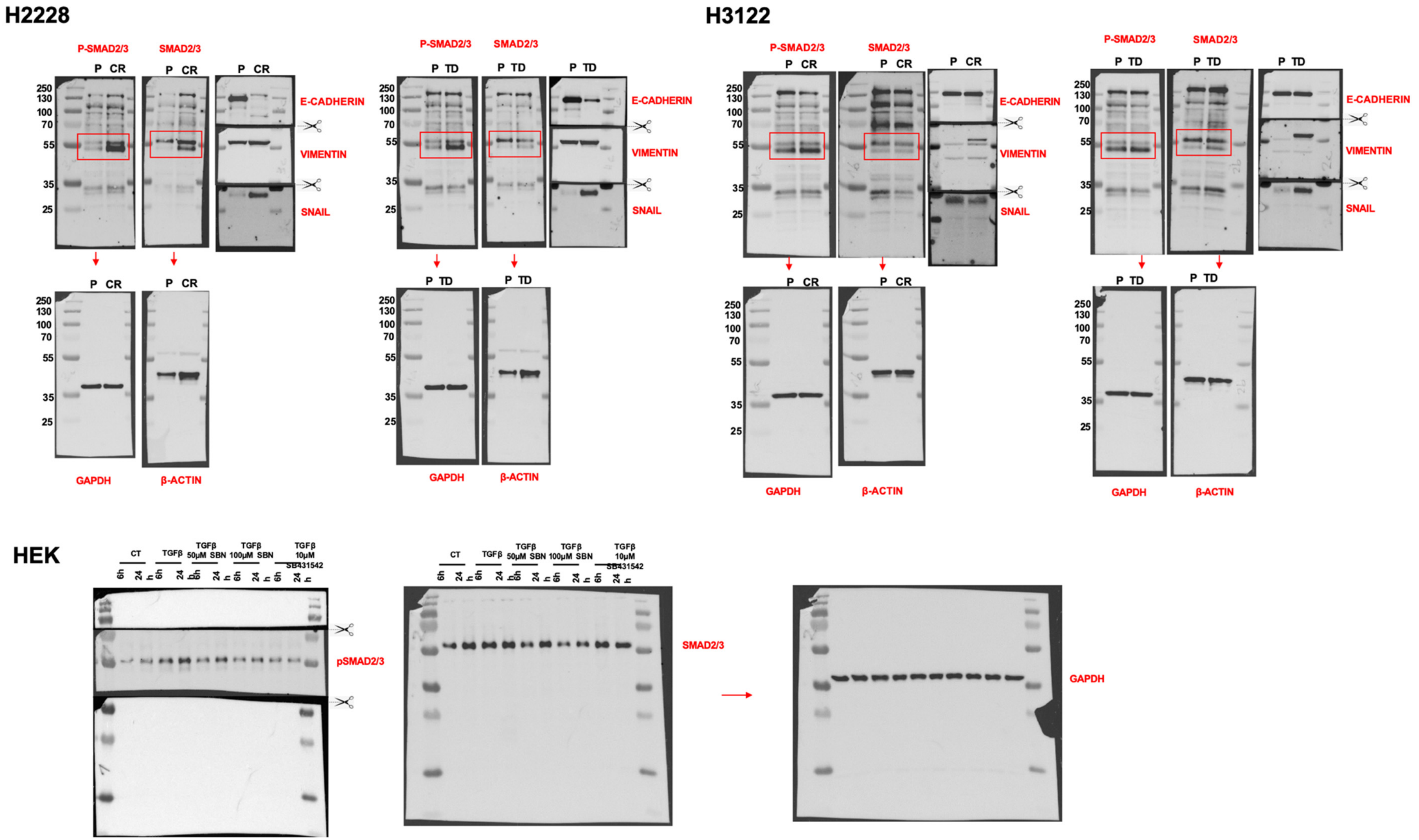

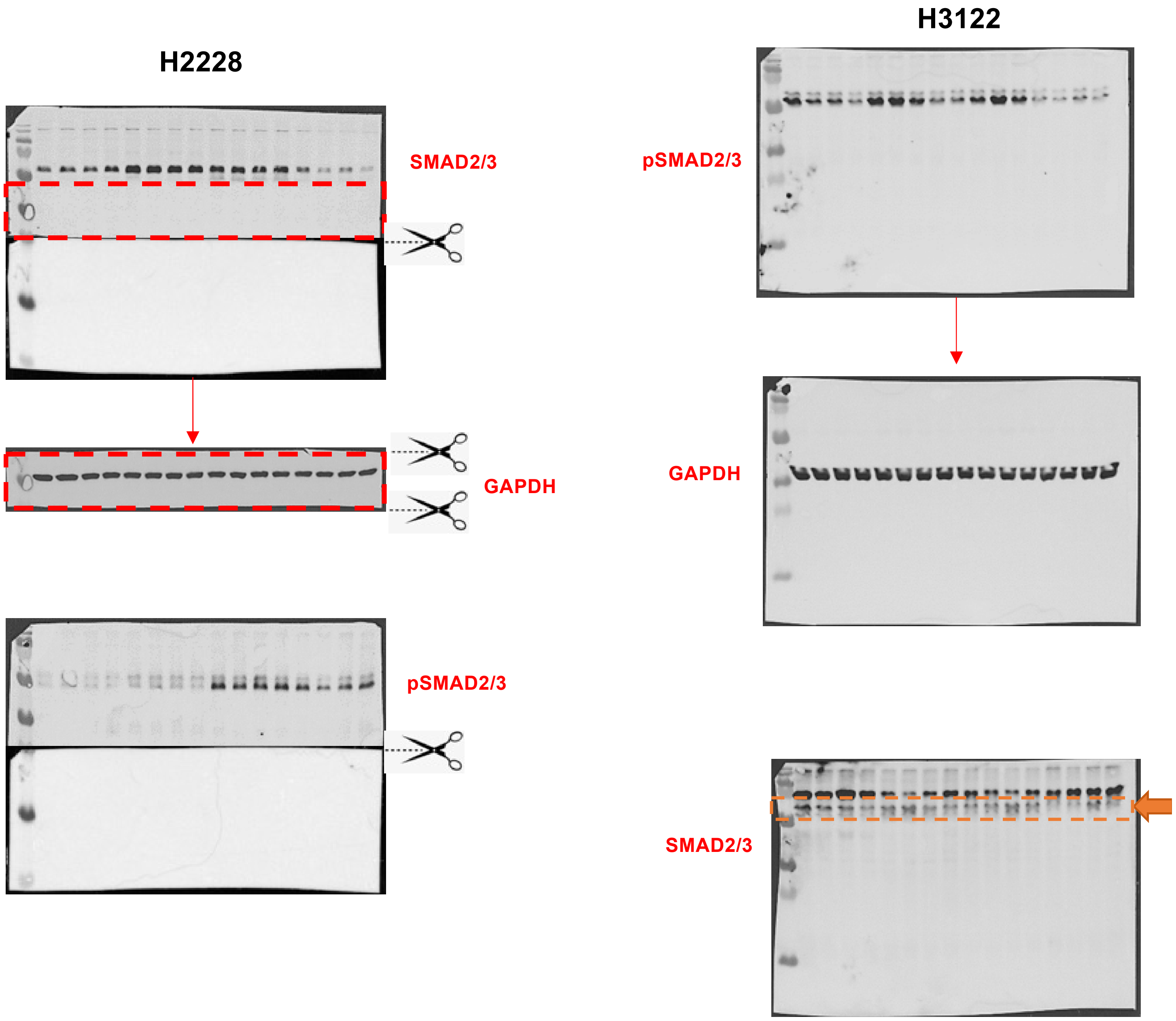
H2228
SMAD2/3
GAPDH
pSMAD2/3
H3122
pSMAD2/3
GAPDH
SMAD2/3
